# Dynamic kirigami structures for integrated solar tracking

**DOI:** 10.1038/ncomms9092

**Published:** 2015-09-08

**Authors:** Aaron Lamoureux, Kyusang Lee, Matthew Shlian, Stephen R. Forrest, Max Shtein

**Affiliations:** 1 Department of Materials Science and Engineering, University of Michigan, Ann Arbor, Michigan 48109, USA; 2 Department of Electrical Engineering and Computer Science, University of Michigan, Ann Arbor, Michigan 48109, USA; 3 School of Art and Design, University of Michigan, Ann Arbor, Michigan 48109, USA

## Abstract

Optical tracking is often combined with conventional flat panel solar cells to maximize electrical power generation over the course of a day. However, conventional trackers are complex and often require costly and cumbersome structural components to support system weight. Here we use kirigami (the art of paper cutting) to realize novel solar cells where tracking is integral to the structure at the substrate level. Specifically, an elegant cut pattern is made in thin-film gallium arsenide solar cells, which are then stretched to produce an array of tilted surface elements which can be controlled to within ±1°. We analyze the combined optical and mechanical properties of the tracking system, and demonstrate a mechanically robust system with optical tracking efficiencies matching conventional trackers. This design suggests a pathway towards enabling new applications for solar tracking, as well as inspiring a broader range of optoelectronic and mechanical devices.

## Abstract (web summary)

Tilting planar photovoltaic panels to track the position of the sun over the day can add to yearly energy consumption. Here, Lamoureaux *et al.* propose a kirigami solar cell structure with solar tracking integrated within the design, reducing cost, structural weight and bulk associated with conventional tracking.

## Graphical abstract

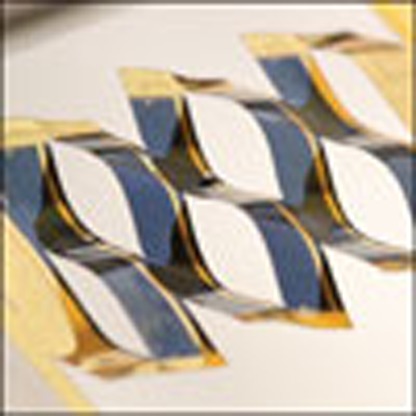

Conventional photovoltaic modules suffer optical coupling losses due to a decrease in projected area that scales with the cosine of the misalignment angle between the cell and the sun (Fig. 1a). To mitigate these losses and maximize power output, flat photovoltaic panels can be tilted to track the position of the sun over the course of the day and/or year. Depending on the geographic location of the system, and whether there are one or two tracking axes, conventional trackers can provide an increase in yearly energy generation between 20 and 40% compared with non-tracking solar arrays^1^,^2^. Furthermore, tracking may be integrated with concentrated photovoltaic systems, where source alignment is critical for maintaining a high concentration factor over a wide range of source angles^3^,^4^,^5^,^6^,^7^,^8^,^9^,^10^,^11^.

Despite the documented effectiveness and relatively mature state of solar tracking, such systems have not been widely implemented due to the high costs, added weight, and additional space required to align the panels, support panel weight, and resist wind loading^12^,^13^. For example, current data indicate that the additional components required for tracking account for ∼12% of the total balance of system costs, a number that is increasing at ∼1% per year^14^. This is in contrast to solar cell and module costs, which continue to decrease rapidly^15^. Furthermore, because of the cumbersome nature of conventional tracking mechanisms, their use has thus far been limited to ground-based and flat-rooftop installations. As a result, residential, pitched rooftop systems, which account for ∼85% of installations, lack conventional tracking options entirely^14^,^16^. To further decrease installation costs and enable new applications, a novel approach to compact and lightweight solar tracking is required.

The principles of origami and kirigami (that is, the folding and cutting of paper, respectively, to achieve a desired shape) have been used in the design of airbags, optical components, stowable spaceborne solar arrays, reprogrammable metamaterials and load-bearing metal structures^17^,^18^,^19^,^20^,^21^. Here, utilizing similar design principles, we show for the first time simple, dynamic kirigami structures integrated with thin-film solar cells that enable highly efficient and macroscopically planar solar tracking as a function of uniaxial strain. For optimized systems, we show tracking to within ±1.0° of the predicted pointing vector, with total power generation approaching that of conventional single-axis tracking systems. Kirigami trackers are also shown to be electrically and mechanically robust, with no appreciable decrease in performance after >300 cycles.

## Results

### Kirigami design principles for integrated solar tracking

**Figure 1:**
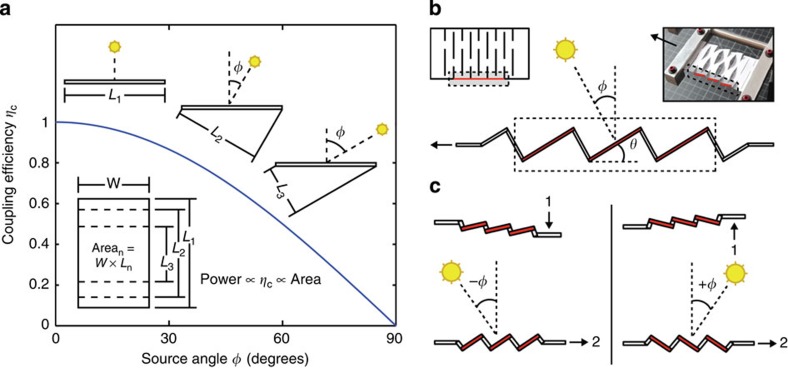
Optical coupling efficiency and novel kirigami trackers. (**a**) Coupling efficiency (*η*_*C*_) versus source angle (*φ*) for a planar solar panel. The panel projected area decreases with the cos*φ*. (**b**) A kirigami tracking structure that, upon stretching, simultaneously changes the angle of the elements comprising the sheet. By incorporating thin-film solar cells into this structure, it may be used as a low-profile alternative to conventional single-axis solar tracking. (**c**) The direction of feature tilt (that is, clockwise or counter-clockwise with respect to the original plane) is controlled by lifting or lowering one end of the sheet (step 1) before the straining process (step 2).

Consider the simple kirigami structure in Fig. 1b, consisting of a linearly cut pattern in an otherwise thin, continuous sheet of flexible material. Pulling on the sheet in the axial direction (that is, perpendicular to the cuts) results in instabilities that produce controlled buckling in the transverse direction (that is, parallel to the cuts), along with a change in feature angle that is synchronized along its length. Importantly, it is possible to control the direction of the feature tilt (that is, clockwise or counter-clockwise with respect to the original plane) by lifting or lowering one end of the sheet before the straining process (Fig. 1c). By integrating similar structures with thin-film solar cells, this unique geometric response may be used as a novel method to accurately track the sun. In practice, these thin-film trackers may be housed inside of thin, double-pane enclosures to ensure weatherproofing and provide support against sagging at larger length scales (Supplementary Fig. 1). To enable dual-axis tracking, the kirigami tracker need simply be rotated about an axis normal to its plane (Supplementary Fig. 2).

**Figure 2:**
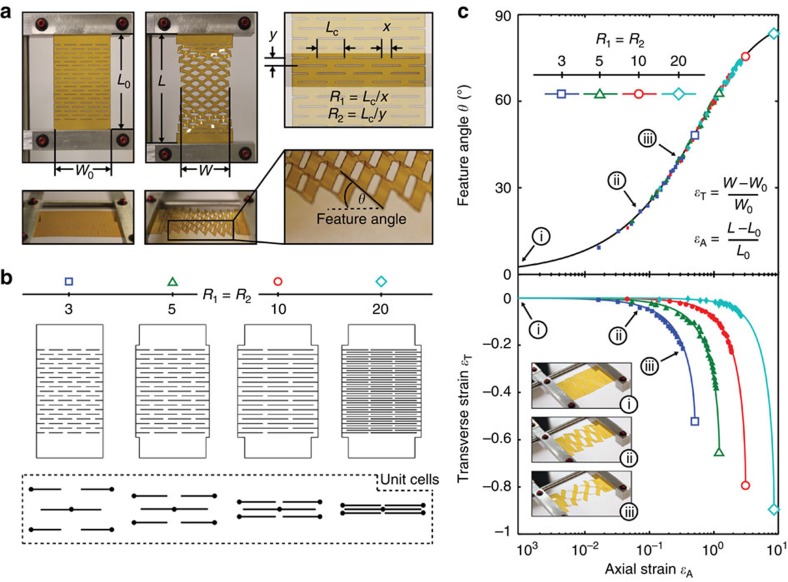
Kirigami cut geometry and geometric system response. (**a**) Response of a Kapton kirigami structure to stretching in the axial direction (*ɛ*_A_) is accompanied by a decrease in sample width (*ɛ*_T_) and a change in feature angle (*θ*). Also shown are the geometric parameters that define the kirigami structure, namely the cut length (*L*_C_) and spacing between cuts in the transverse (*x*) and axial (*y*) directions, which can be expressed in terms of the dimensionless parameters, *R*_1_ and *R*_2_. (**b**) Schematics of four kirigami structures, where *R*_1_=*R*_2_=3, 5, 10 and 20, along with their corresponding units cells. (**c**) *ɛ*_T_ and *θ* versus *ɛ*_A_ for several kirigami structures where *R*_1_=*R*_2_=3, 5, 10 and 20 (**b**). Theoretical predictions per equations (1) and (2) are shown by solid lines, while the closed symbols represent experimental data from a 50 μm-thick Kapton sample of the appropriate geometry. While larger *R*_1_ and *R*_2_ enable increased axial strains and correspondingly larger transverse strains, the change in feature angle is independent of cut geometry.

The geometric response of a simple kirigami structure is clarified for a Kapton sheet tracker in Fig. 2a, where the kirigami geometry is defined by the cut length (*L*_C_), as well as the spacing between cuts in the transverse (*x*) and axial (*y*) directions. The change in feature angle (*θ*) and decrease in sample width (that is, transverse strain, *ɛ*_T_) as a function of stretching (that is, axial strain, *ɛ*_A_) are:


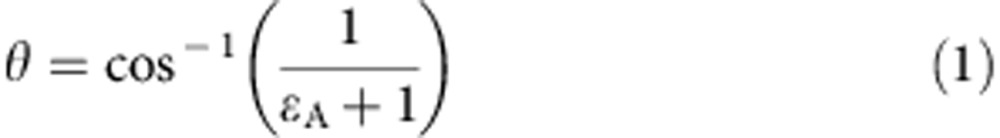



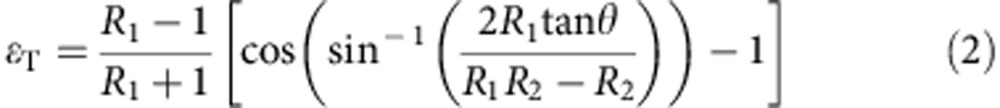


where 
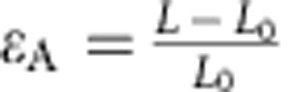
, 
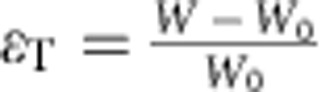
 and 
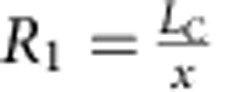
 and 
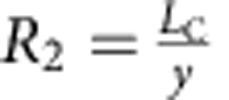
 are dimensionless parameters (Supplementary Note 1). To examine the effect of system geometry on response, *R*_1_ and *R*_2_ were systematically varied such that *R*_1_=*R*_2_=3, 5, 10 and 20, as shown in Fig. 2b. The response characterized by equations (1) and (2) (solid lines) is experimentally verified (closed symbols) in Fig. 2c for each kirigami structure. We find that larger *R*_1_ and *R*_2_ enable greater *ɛ*_A_ and correspondingly greater *ɛ*_T_. A plot of *θ* versus *ɛ*_A_ confirms that the dependence of *θ* on *ɛ*_A_ is independent of cut geometry. For the samples tested, *θ* was controlled to within ±1.0° of its value in equation (1). Also shown are the pseudo-plastic limits for each superstructure, where the maximum axial strains and corresponding maximum feature angles, *θ*_MAX_, are depicted as open symbols. For each structure, *θ*_MAX_ is solely dependent on cut geometry, setting the upper limit of tracking without shadowing in the axial direction. That is:


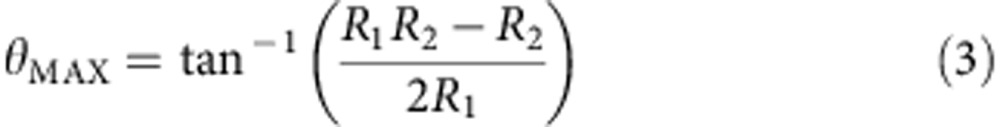


### Optical properties and optimized tracking performance

**Figure 3:**
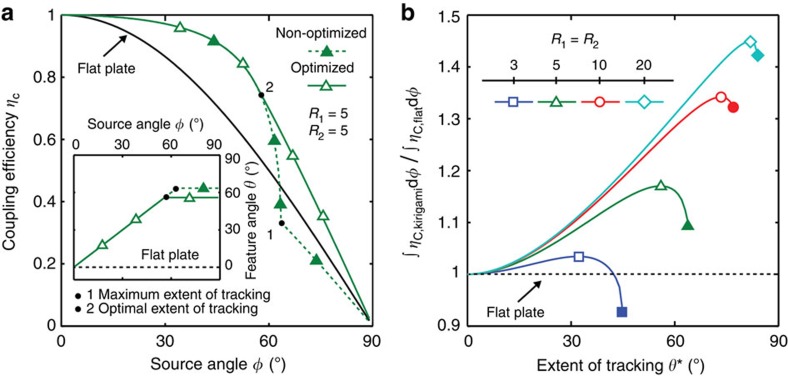
Optimization of tracking process to maximize coupling efficiency. (**a**) Coupling efficiency (*η*_*C*_) versus source angle (*φ*) for two systems with different extent of tracking (θ*). *Inset:* Feature angle (*θ*) versus *φ*. Non-optimized tracking (closed symbols) close to the geometric maximum (*θ*_MAX_, point 1) results in a sharp decrease in sample width that decreases optical coupling efficiency. Instead, coupling efficiency is optimized (open symbols) by a tradeoff between sample narrowing, self-shadowing, and cosine losses (c.f. equation (4) in text) corresponding instead to an optimal angle (point 2). Simulated system response is shown for *R*_1_=*R*_2_=5, and is compared to a conventional non-tracking panel. (**b**) Coupling efficiency, *η*_*C*_ integrated over a range of tracking angle (from *φ*=0 to *φ*=θ*) and normalized to conventional planar cell performance. For a given kirigami structure, optimal performance is obtained by tracking the source at normal incidence to θ* corresponding to the maximum of each curve. For comparison, tracking to *θ*_MAX_ versus tracking to the optimal θ* is shown as solid and open symbols, respectively.

Remarkably, this simple structure is very effective for solar tracking, which we quantify as follows. We define the total optical coupling efficiency (*η*_C_) as a function of individual optical coupling losses in the axial (*η*_A_) and transverse (*η*_T_) directions, as well as cosine losses (*η*_O_) and surface reflection (*η*_R_) at oblique incident angles. For an optical source moving in the plane normal to the tracking axis, and assuming a suitable anti-reflective coating, *η*_C_ is simply:


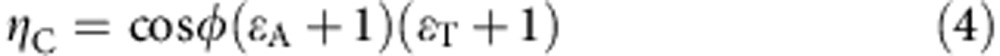


where *φ* is the source angle from the normal to the module plane (Fig. 1a), and *ɛ*_A_ and *ɛ*_T_ are the axial and transverse strains, respectively (Supplementary Note 2). To maximize *η*_C_, shadowing in the axial direction, as well as cosine losses, must be balanced against the decrease in the width resulting from the geometric response of the structure. Indeed, tracking to *θ*_MAX_ may not be optimal due to the sharp decrease in projected area beyond some critical strain (Fig. 2c). This geometric subtlety is quantified in Fig. 3a, where we analyze the differences in performance for optimized tracking (open symbols), and one variation of non-optimized tracking to *θ*_MAX_ defined by equation (3) (closed symbols).

As shown in the inset of Fig. 3a (*θ* versus *φ*), each structure tracks the source as characterized by a unity slope, until a predetermined value of θ* is reached (that is, extent of tracking), after which the angle of the structure is held constant. Here, θ* is denoted as point 1 and point 2 for non-optimized and optimized tracking, respectively. The effects of these tracking modes are shown in the plot of *η*_C_ versus *φ*, as defined by equation (4). Note the difference in *η*_C_ at large values of *φ*. Whereas tracking to *θ*_MAX_ (closed symbols) causes a large decrease in sample width and *η*_C_ near the geometric limits of the structure, optimized tracking (open symbols) minimizes the tradeoff between sample narrowing (*η*_T_), self-shadowing (*η*_A_) and cosine losses (*η*_O_). Figure 3b shows the extension of this analysis to other cut geometries, where *η*_C_ is integrated over a range of tracking angles (from *φ*=0 to *φ*=θ*) and normalized to conventional planar cell performance under identical operating conditions. Figure 3b provides the appropriate tracking procedure for a given kirigami structure, where optimal performance is obtained by tracking the source at normal incidence until reaching the θ* corresponding to the maximum of each curve (for *R*_1_=*R*_2_=3, θ*≈37°, for *R*_1_=*R*_2_=5, θ*≈54°, for *R*_1_=*R*_2_=10, θ*≈73°, and for *R*_1_=*R*_2_=20, θ*≈82°). For comparison, tracking to *θ*_MAX_ versus the optimized tracking limit are shown as solid and open symbols, respectively.

### Fabrication of GaAs kirigami tracker

Due to the buckling of the sheet along the transverse direction, it is advantageous to combine this tracking design with solar cells that can similarly flex to accommodate the non-planarity of the sheet. Hence, we mounted thin (∼3 μm), flexible, crystalline gallium arsenide (GaAs) photovoltaic cells with the kirigami tracking structure in Fig. 2. The cells were fabricated by a combination of non-destructive (and thus potentially low cost) epitaxial lift-off (ELO) and cold welding between gold films pre-deposited on both the backside of the GaAs solar cells and on the Kapton structure using procedures described previously^22^,^23^,^24^. Notably, the solar cells were patterned to coincide with predetermined values of *R*_1_ and *R*_2_ to minimize edge recombination losses as well as prevent damage during the laser dicing cell singulation process. Finally, a bilayer anti-reflective coating consisting of TiO_2_ and MgF_2_ was used to minimize reflection losses at oblique angle incidence resulting from the bowing of the kirigami cells during stretching (Supplementary Fig. 3).

### Validation of tracking performance

**Figure 4:**
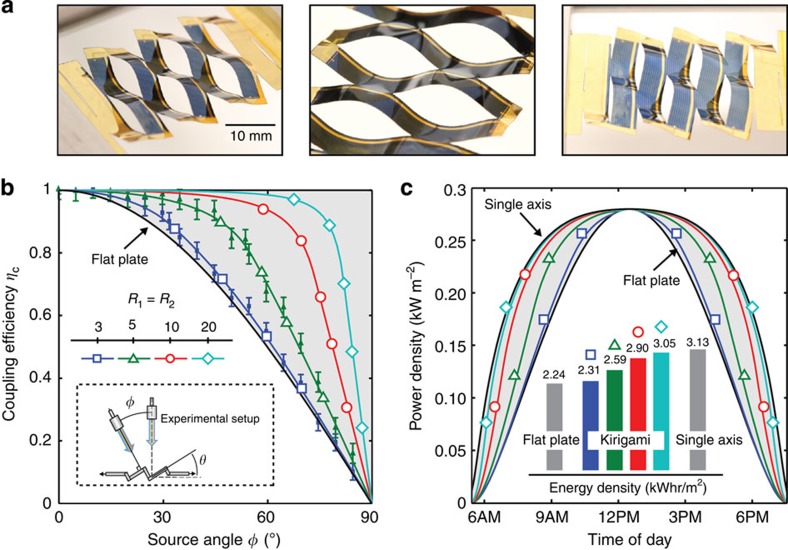
Tracking performance for GaAs kirigami trackers. (**a**) Integrated thin-film, crystalline GaAs solar cells, mounted by cold weld bonding on a Kapton carrier substrate, as used for testing. Here, *L*_C_=15 mm, *x*=5 mm and *y*=5 mm (*R*_1_=*R*_2_=3). (**b**) Normalized solar cell short circuit current density *J*_SC_(*φ*)/*J*_SC_(*φ=*0) for two samples, where *R*_1_=*R*_2_=3 and *R*_1_=*R*_2_=5 (closed symbols). Also shown are the simulated data for coupling efficiency (*η*_C_) obtained from equation (4) (solid lines, open symbols). The agreement between experimental and simulated results suggests that *η*_*C*_ is a direct measure of optical coupling, and that performance may be optimized by increasing *R*_1_ and *R*_2_. (**c**) Output electrical power density incident on the solar cell versus time of day for several kirigami cut structures, stationary panel and single-axis tracking systems in Phoenix, AZ (33.45° N, 112.07° W) during the summer solstice. *Inset:* Integration of the curves yields the associated energy densities, where kirigami-enabled tracking systems are capable of near single-axis performance.

An example kirigami tracker with >99% GaAs solar cell coverage is shown in Fig. 4a, where *R*_1_=*R*_2_=3. Each tracker was systematically strained to follow a moving AM1.5G collimated light source, and the solar cell current density versus voltage (*J–V*) characteristics were obtained as a function of illumination angle, *φ*. A schematic of this experiment is shown in the inset of Fig. 4b, where the kirigami tracker was strained to track the light source to the optimal θ* per the process shown in Fig. 3. Figure 4b plots the ratio of the normalized angle-dependent short circuit current density (*J*_SC_(*φ*)/*J*_SC_(*φ=0*)) for two samples, where *R*_1_=*R*_2_=3 and *R*_1_=*R*_2_=5 (closed symbols). Also shown is *η*_C_ defined by equation (4) for several cut geometries (open symbols, solid lines). As expected, larger *R*_1_ and *R*_2_ lead to an increase in *η*_C_ due to the suppression of *ɛ*_T_ at equivalent *ɛ*_A_. Furthermore, *J*_SC_(*φ*)/*J*_SC_(*φ=0*) matches *η*_C_ predicted by equation (4), suggesting that *η*_C_ is a direct measure of optical coupling in the presence of a suitable cell anti-reflective coating.

The effect of kirigami geometry on power generation is compared with fixed planar cells and conventional single-axis systems in Fig. 4c for Phoenix, AZ (33.45° N, 112.07° W) during the summer solstice. The power conversion efficiency for each system was assumed to be 20%. As *R*_1_ and *R*_2_ increase, the system becomes more efficient in the morning and evening at angles far from the zenith, and the output power density increases accordingly. As shown in the inset, the output energy density for an optimized kirigami system approaches that of conventional single-axis tracking in the limit of large values of *R*_1_ and *R*_2_. This is indeed remarkable for a solar cell that remains essentially flat and without a change in macroscopic orientation. For more information on system response (that is, *θ* and *η*_C_ versus time of day for *R*_1_=*R*_2_=10), see Supplementary Fig. 4.

### Effect of mechanical stresses on device performance

The electrical and mechanical responses to strain and cycling were also considered with implications for long-term solar tracking. For the Kapton/GaAs tracker where *R*_1_=*R*_2_=5, there was no systematic change in either fill factor (*FF*) or open circuit voltage (*V*_OC_) from *θ*=0 to θ* (Supplementary Fig. 5a), with repeated measurements over 350 cycles yielding similar results (Supplementary Fig. 5b). Over a similar range, strain energy (that is, the energy stored in the system undergoing deformation) was shown to decrease by ∼36%, without failure, due to plastic deformation and crack propagation at the cuts for high strains. By optimizing the cut geometry and thus minimizing stress at the cuts, it is possible to significantly decrease strain fade—for example, in comparable Kapton kirigami trackers where *R*_1_=*R*_2_=3, 5 and 10, the strain energy was shown to decrease by ∼74, ∼17 and only ∼3%, respectively, over 1,000 cycles. Other substrates with improved mechanical and thermal stabilities (for example, spring steel) are also currently being investigated as more robust materials platforms with longer operational lifetimes.

## Discussion

In summary, we have shown that kirigami structures combined with thin-film active materials may be used as a simple, low-cost, lightweight and low-profile method to track solar position, thereby maximizing solar power generation. These systems provide benefits over conventional mechanisms, where additional heavy, bulky components and structural supports are often required to synchronize tracking between panels and accommodate forces due to wind loading. By eliminating the need for such components, kirigami serves to decrease installation costs and expose new markets for solar tracking, including widespread rooftop, mobile and spaceborne installations. Kirigami-enabled systems are also cost-effective and scalable in both fabrication and materials, and similar design rules may be extended for use in a wide range of optical and mechanical applications, including phased array radar and optical beam steering.

## Methods

### Fabrication of thin-film GaAs solar cells

Epitaxial layers of *p–n* junction gallium arsenide (GaAs) active material on an AlAs sacrificial layer were grown by gas-source molecular beam epitaxy on a 2-inch diameter (100) GaAs substrate. For the ND-ELO process, 0.2 μm thick GaAs buffer layer followed by a 20-nm thick AlAs sacrificial layer were grown, first. Then, following inverted photovoltaic device layers were grown: 0.1 μm thick, 5 × 10^18^ cm^−3^ Be-doped GaAs *p-*contact layer, 0.025 μm thick, 2 × 10^18^ cm^−3^ Be-doped Al_0.20_In_0.49_Ga_0.31_P window layer, 0.15 μm thick, 1 × 10^18^ cm^−3^ Be-doped *p*-GaAs emitter layer, 3.0 μm thick, 2 × 10^17^ cm^−3^ Si-doped *n*-GaAs base layer, 0.05 μm thick, 6 × 10^17^ cm^−3^ Si-doped In_0.49_Ga_0.51_P back surface field layer, and 0.05 μm thick, 5 × 10^18^ cm^−3^ Si-doped *n-*GaAs contact layer. The sample was then coated with a 300 nm thick Au layer by e-beam evaporation, and bonded to a 50 μm-thick E-type Kapton sheet (also coated in 300 nm Au layer) using cold weld bonding by applying a pressure of 4 MPa for 3 min at a temperature of 200 °C. After bonding, the photovoltaic epitaxial active region and Kapton carrier were isolated from the bulk wafer using ELO by selectively removing the AlAs sacrificial layer in dilute (15%) hydrofluoric acid (HF) solution at room temperature. After ND-ELO, a Pd(5 nm)/Zn(20 nm)/Au(700 nm) front metal contact was patterned using photolithography. Then, the device mesas were similarly defined using photolithography and subsequent chemical etching using H_3_PO_4_:H_2_O_2_:deionized H_2_O (3:1:25). The exposed, highly Be-doped 150 nm thick *p*+ GaAs contact layer was selectively removed using plasma etching. After annealing the sample for 1 h at 200 °C to facilitate ohmic contact formation, the sidewalls were passivated with 1-μm thick polyimide applied by spin coating. After curing the sample at 300 °C for 30 min, the polyimide was selectively removed by photolithography and plasma etching. The external contact pad was patterned with Ti (10 nm)/Au (500 nm). Finally, a bilayer anti-reflection coating consisting of TiO_2_ (49 nm) and MgF_2_ (81 nm) was deposited by e-beam evaporation.

### Fabrication of kirigami trackers

Kapton kirigami structures were fabricated using a 50 W Universal Laser Systems CO_2_ laser (2% power, 2.5% speed, 500 pulses per inch (PPI)). Following the schematics in Fig. 2b, the following cut dimensions were used; *R*_1_=*R*_2_=3 (*L*_C_=6 mm, *x*=2 mm and *y*=2 mm), *R*_1_=*R*_2_=5 (*L*_C_=10 mm, *x*=2 mm and *y*=2 mm), *R*_1_=*R*_2_=10 (*L*_C_=20 mm, *x*=2 mm and *y*=2 mm), *R*_1_=*R*_2_=20 (*L*_C_=20 mm, *x*=1 mm and *y*=1 mm). Kapton/GaAs solar trackers were fabricated using the ND-ELO process described previously, and then cut using an X-ACTO knife. As outlined in the main text, two samples were used, namely *R*_1_=*R*_2_=3 (*L*_C_=15 mm, *x*=5 mm and *y*=5 mm) and *R*_1_=*R*_2_=5 (*L*_C_=15 mm, *x*=3 mm and *y*=3 mm). It should be noted that, for the cycling *J–V* measurements, the GaAs was patterned only along the dimension *x* (Supplementary Note 2) to eliminate the effect of *ɛ*_T_ and *η*_C_ on electrical response (that is, to ensure a constant *J*_SC_ and comparable *J–V* curves).

### Measurement of axial and transverse strain

The Kapton kirigami structures described previously were systematically strained to the maximum feature angle, *θ*_MAX_ (equation (3)), using a homemade micro-strain apparatus. The straining process was imaged *in situ* using two cameras: one facing directly downwards to capture *ɛ*_T_ and a second facing the edge of the sample to capture *θ* (Fig. 2a). Both cameras captured the axial strain, *ɛ*_A_. The resulting images were analysed using ImageJ (W.S. Rasband, US National Institutes of Health, Bethesda, Maryland, USA), where a global calibration scale was used to define measurement lengths. It should be noted that, in some cases, limitations imposed by the range of motion of the apparatus prohibited data collection at high strain values, as shown in Fig. 2c.

### Electrical characterization of GaAs kirigami trackers

The Kapton/GaAs kirigami trackers described previously were systematically strained using a micro-strain apparatus to track a moving AM1.5G light source (Oriel solar simulator, model 91191 with Xenon arc lamp and AM 1.5 global filter, simulated 1 sun, 100 mW cm^−2^ intensity), following the optimal tracking process described in Fig. 3 (that is, *θ*=0° to *θ*=θ*). The *J–V* characteristics (that is, *J*_SC_, *V*_OC_ and FF) were measured at each angle using a semiconductor parameter analyser (SPA, Agilent 4155B), in increments of five degrees, from normal incidence (*φ*=0°) to *φ*=90°. To determine the effect of cycling on cell performance, the solar tracker was repeatedly strained to *θ*=θ*, while the source was kept constant at *φ*=0°. Cell performance (that is, *J*_SC_, *V*_OC_ and *FF*) was measured every 10 cycles, when *θ*=0 (and consequently *φ*=0°), for 350 cycles (to simulate ∼1 year of operation), as shown in Fig. 4d.

### Mechanical characterization of kirigami trackers

The stress–strain characteristics of the Kapton and Kapton/GaAs kirigami trackers described previously were measured using a TA.XT*Plus* Texture Analyser (Texture Technologies, Hamilton, Massachusetts, USA) and the *Exponent* (Texture Technologies, Hamilton, Massachusetts, USA) software package. For the Kapton trackers, the sample length as measured from the first cut to the last cut in the axial direction was 36 mm. For the Kapton/GaAs trackers, the sample length as measured from the first cut to the last cut in the axial direction was 33 mm. Each sample was strained per the optimized tracking process described in Fig. 3, and the resulting stress–strain behaviour was recorded. This process was repeated 1,000 times, and the resulting curves were integrated to find the strain energy. The strain fade was calculated as the percentage difference in strain energy between cycle 1 and cycle 1,000 (that is, 
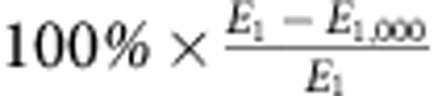
).

## Additional information

**How to cite this article:** Lamoureux, A. *et al.* Dynamic kirigami structures for integrated solar tracking. *Nat. Commun.* 6:8092 doi: 10.1038/ncomms9092 (2015).

## Supplementary Material

Supplementary Information Supplementary Figures 1-5 and Supplementary Notes 1-2
