# Supplementary material for: Dynamic kirigami structures for integrated solar tracking
Source: Nat Commun. 2015 Sep 8;6:8092. doi: 10.1038/ncomms9092 (PMC4569711; doi:10.1038/ncomms9092)
Supplement: Supplementary Information — Supplementary Figures 1-5 and Supplementary Notes 1-2 [file ncomms9092-s1.pdf]

Supplementary Figure 1

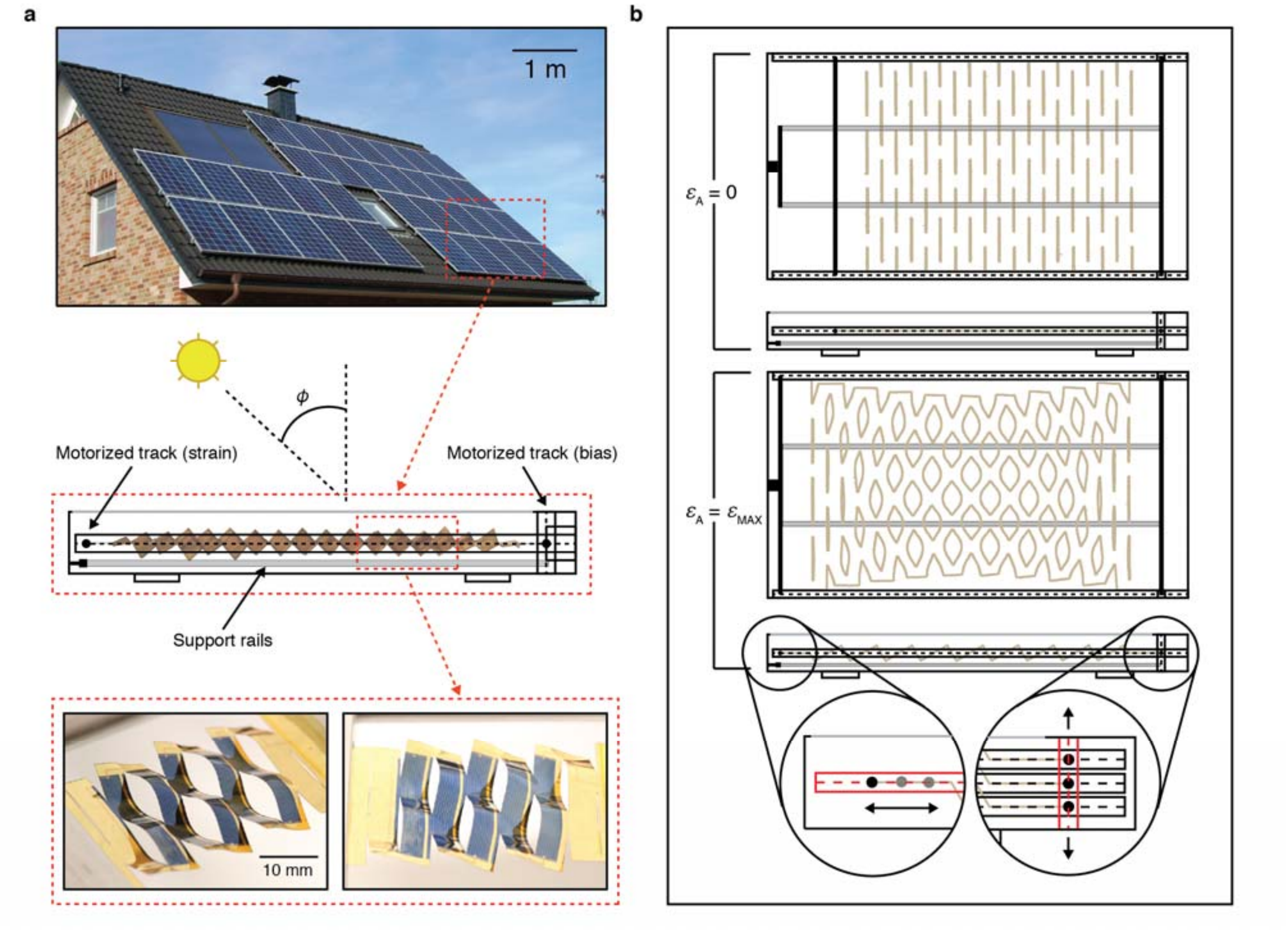

Rooftop kirigami tracking and schematics of module. (a) Thin-film kirigami constructs may be integrated with low-profile, rigid enclosures to ensure weatherproofing and compatibility with existing rooftop installation hardware. Tensioned support cables may be used in large arrays support system weight and prevent sagging of the structure. **(b)** Schematic view of kirigami tracking system when $\varepsilon_A = 0$ and also maximum strain (i.e. $\varepsilon_A = \varepsilon_{MAX}$). Note the detailed views of each motorized track, which are used to provide strain and vertical biasing during operation.

Supplementary Figure 2

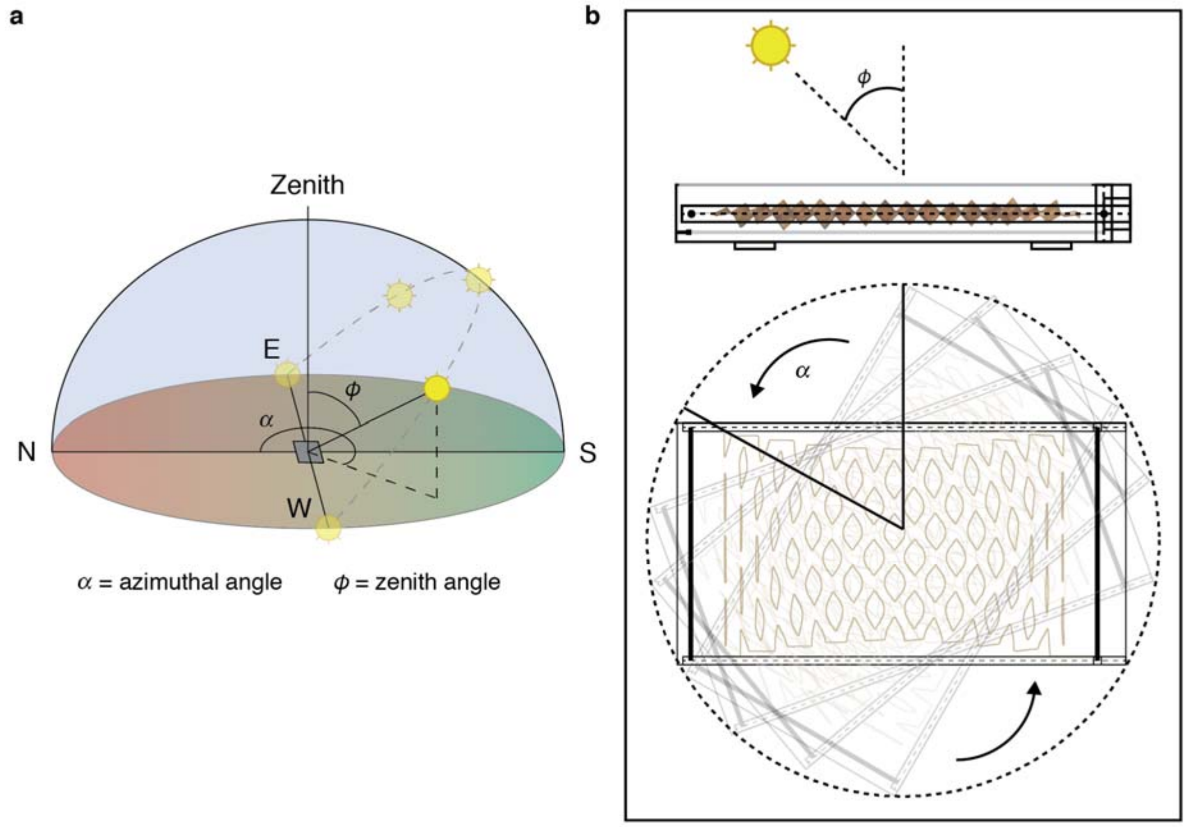

Dual-axis tracking using kirigami tracker. (a) The azimuthal (α) and zenith (ϕ) angles fully characterize the trajectory of the sun as a function of time of day, time of year, and geographic location. **(b)** To enable dual-axis tracking, the proposed kirigami tracker need simply be rotated about its base to track the azimuth of the sun.

Supplementary Figure 3

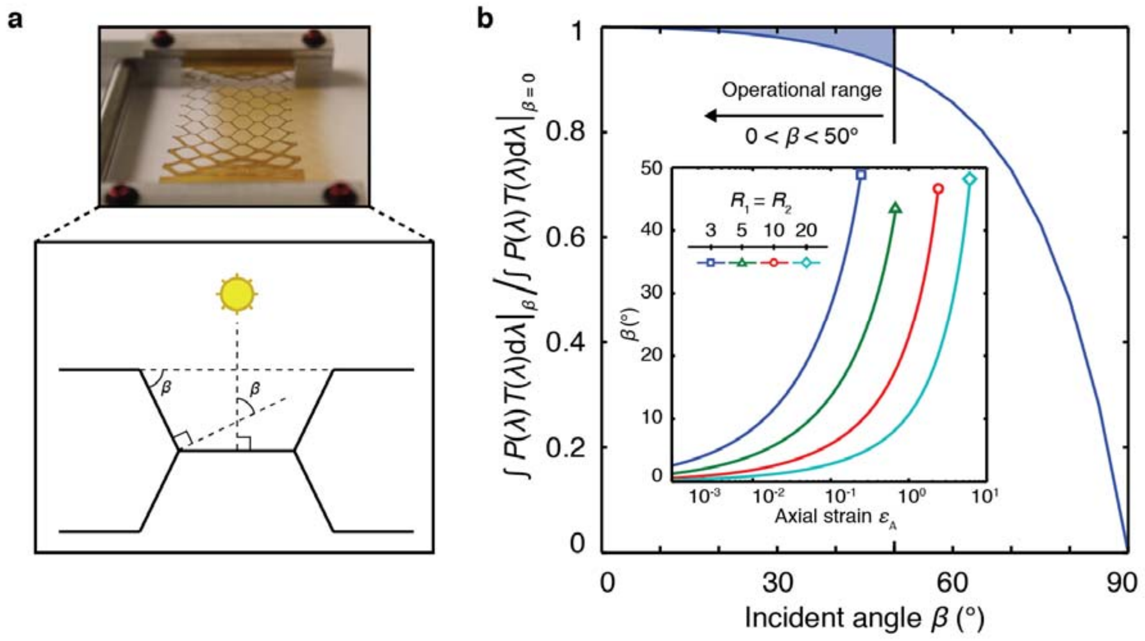

Surface reflection losses and anti-reflection coating. (a) Due to the bowing of the kirigami tracker out of the original plane of the sheet, we must account for oblique angle incidence and any reflection losses as a function of angle, *β*. **(b)** Normalized transmitted power integrated from 350 to 900 nm *vs.* *β* for the bilayer ARC (49 nm TiO_2 and 81 nm MgF_2) used in this study. Incident power, *P*(λ), was taken as AM1.5G, and transmission, *T*(λ), was calculated using common transfer matrix methods. *Inset:* *β* *vs.* axial strain, ε_A, for $R_1 = R_2 = 3$, 5, 10, and 20. Interestingly enough, while each sample tracks to a different maximum ε_A (according to the principles of optimal tracking outlined in Fig. 3), *β* at ε_A (max) remains consistent, and below 50°. Thus, we conclude 0°< *β* <50° signifies the operating range for tracking, over which normalized transmission is shown to remain relatively constant.

Supplementary Figure 4

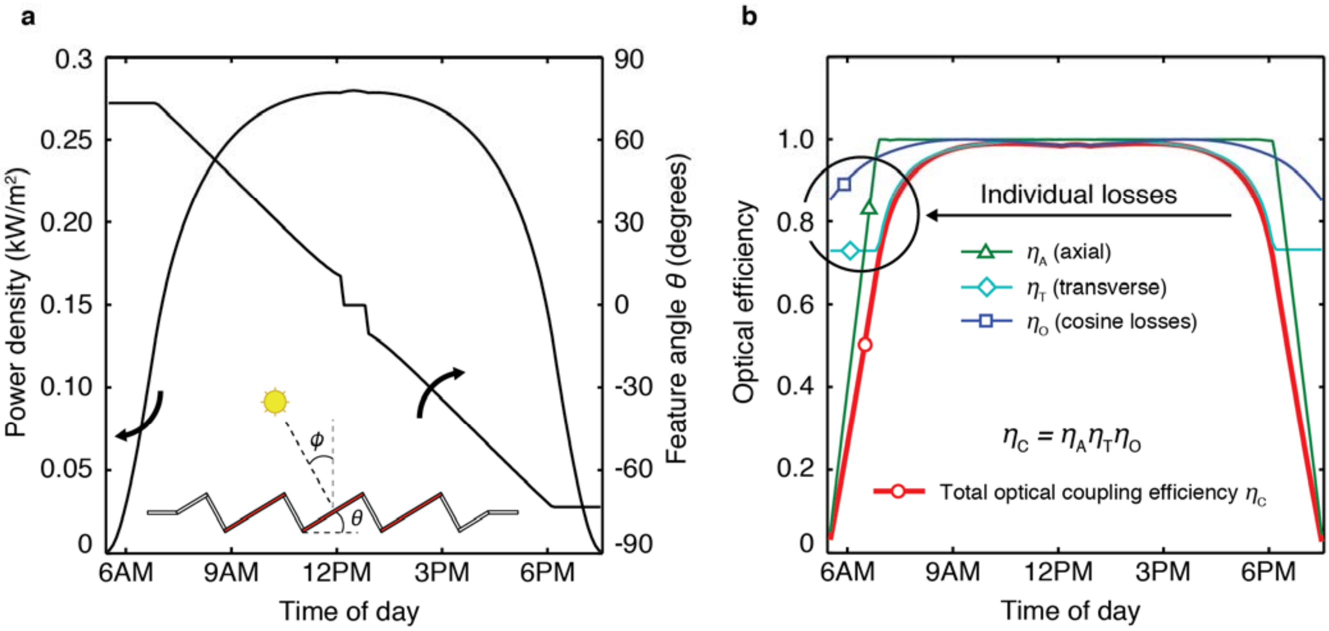

Additional system response and optical efficiency breakdown. (a) Power density and feature angle, θ, *vs.* time of day for a kirigami tracking structure in Phoenix, AZ (33.45° N, 112.07° W) during the summer solstice, where $R_1 = R_2 = 5$. PCE was assumed to be 20%. **(b)** Individual optical loss mechanisms for a kirigami tracking structure in Phoenix, AZ during the summer solstice, where $R_1 = R_2 = 5$. The total optical efficiency is also shown.

Supplementary Figure 5

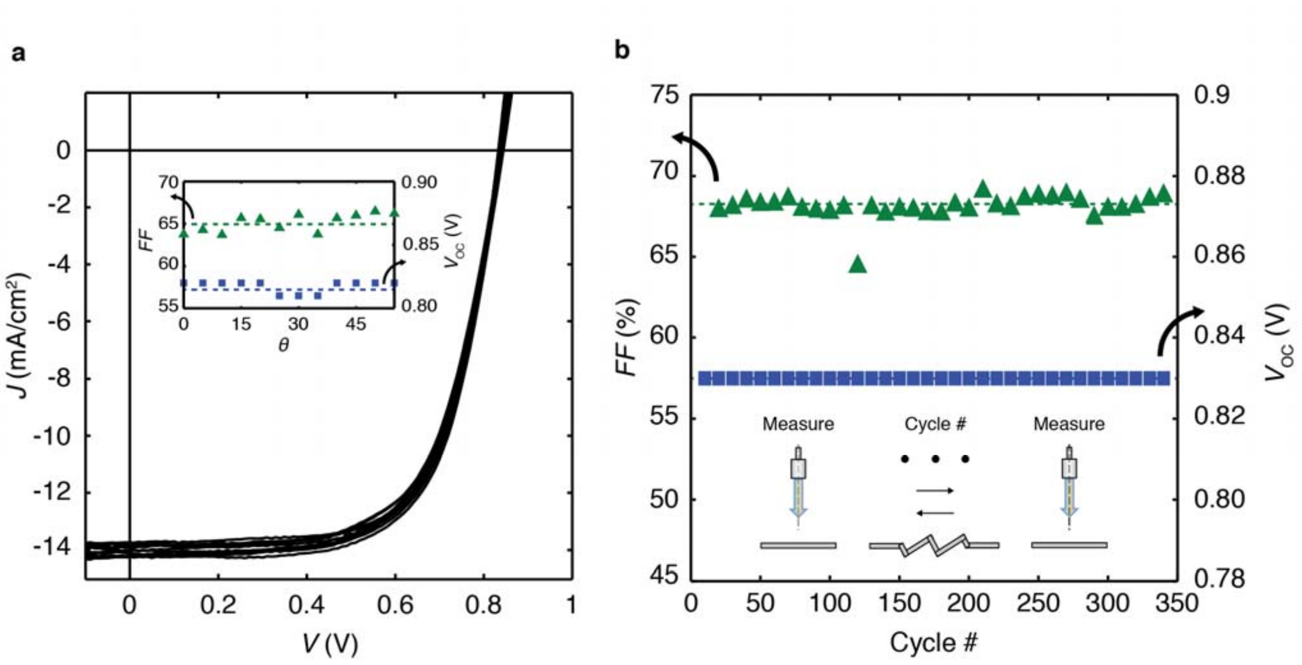

Effect of cycling on electrical response for kirigami tracker. (a) J-V characteristics for a Kapton®/GaAs tracker where $R_1 = R_2 = 5$ at incremental angles of simulated AM1.5G incidence at 1 sun (100 mW/cm^2) intensity, oriented normal to the surface of the solar cells (i.e. $\phi = \theta$). Inset: Fill factor, *FF*, and open circuit voltage, V_{OC}, show no appreciable degradation up until the optimal θ*, and the variation as shown (±1.1 and ±4.5 mV for *FF* and V_{OC}, respectively) is attributed to the error in control over feature angle (±1.0°, as noted earlier in the text). The power conversion efficiency of these devices under 1 sun AM1.5G illumination was 7.4 ± 0.1% at the outset, with virtually no degradation upon cycling. **(b)** *FF* and V_{OC} *vs.* cycle number for a Kapton®/GaAs tracker, where $R_1 = R_2 = 5$. There was no systematic change in *FF* or V_{OC}, with the random variation in measurements due to error in control over feature angle.

Supplementary Note 1

Derivation of geometric response

Feature angle, θ

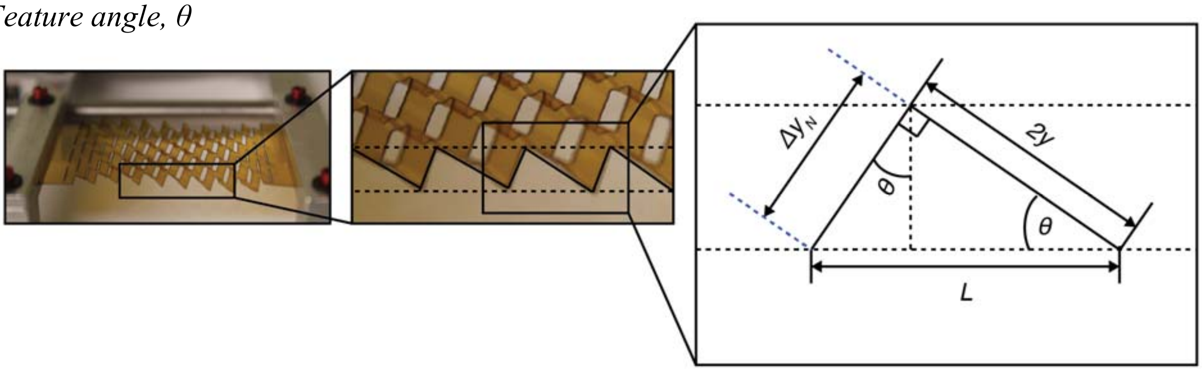

$$L_0 = 2y \quad (1)$$

$$L = \frac{2y}{\cos\theta} \quad (2)$$

$$\Delta y_N = 2y\tan\theta \quad (3)$$

$$\varepsilon_A = \frac{L - L_0}{L_0} = \frac{1}{\cos\theta} - 1 \quad \rightarrow \quad \theta = \cos^{-1}\left(\frac{1}{\varepsilon_A + 1}\right) \quad (4)$$

Transverse strain, ε_T

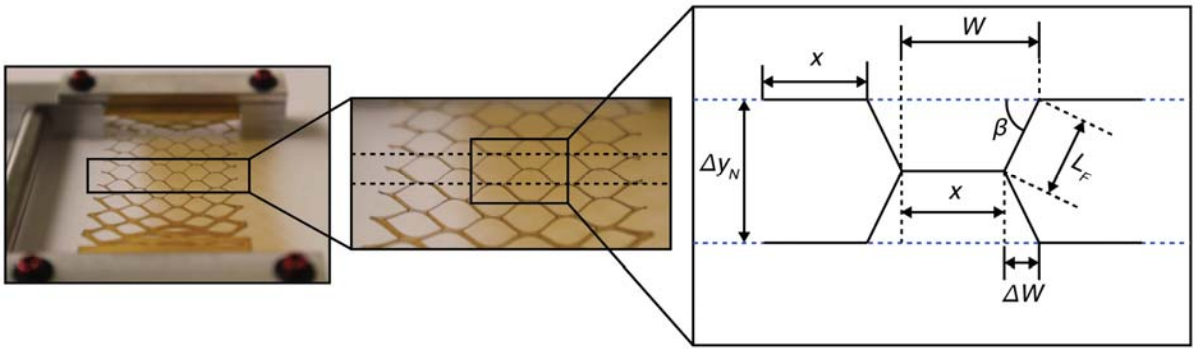

$$W_0 = x + L_F \quad (5)$$

$$W = x + \Delta W \quad (6)$$

$$\varepsilon_T = \frac{W - W_0}{W_0} = \frac{L_F\cos\beta - L_F}{x + L_F} \quad (7)$$

where:

$$\Delta W = L_F \cos\beta \tag{8}$$

$$\beta = \sin^{-1}\left(\frac{y\tan\theta}{L_F}\right) \tag{9}$$

$$L_F = \frac{L_C - x}{2} \tag{10}$$

Substituting ΔW, β, and L_F and using $R_1 = L_C/x$ and $R_2 = L_C/y$ to non-dimensionalize, simplification of ε_T yields:

$$\varepsilon_T = \frac{R_1 - 1}{R_1 + 1}\left[\cos\left(\sin^{-1}\left(\frac{2R_1\tan\theta}{R_1 R_2 - R_2}\right)\right) - 1\right] \tag{11}$$

Supplementary Note 2

Derivation of coupling efficiency, η_C

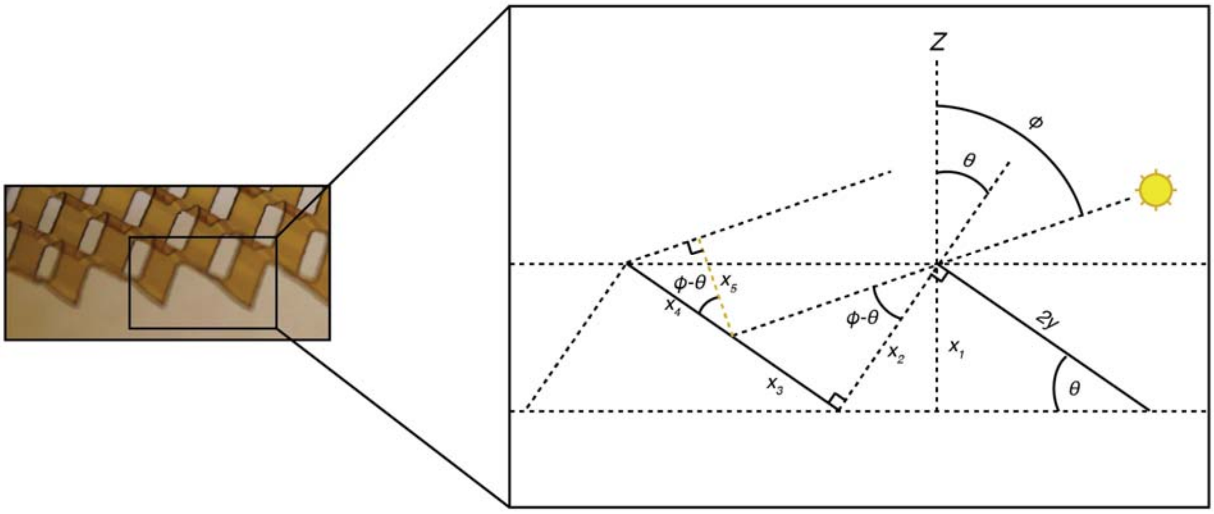

To determine the total coupling efficiency, η_C, we must account for individual optical coupling efficiencies in the axial (η_A) and transverse (η_T) directions, as well as cosine losses (η_O) and surface reflection (η_R) at oblique incident angles:

$$\eta_C = \eta_A \eta_T \eta_O \left(1 - \eta_R\right) \quad (12)$$

In the presence of a suitable anti-reflective coating (ARC) (such as the TiO_2/MgF_2 bilayer ARC used in this paper, see Supplementary Fig. 3), η_R becomes negligible such that:

$$\eta_C = \eta_A \eta_T \eta_O \quad (13)$$

Using the supplemental figure above to determine η_A:

$$x_1 = 2y\sin\theta \quad (14)$$

$$x_2 = \frac{x_1}{\cos\theta} = 2y\tan\theta \quad (15)$$

$$x_3 = x_2\tan\left(\phi - \theta\right) = 2y\tan\theta\tan\left(\phi - \theta\right) \quad (16)$$

$$x_4 = 2y - x_3 = 2y - 2y\tan\theta\tan\left(\phi - \theta\right) = 2y\left[1 - \tan\theta\tan\left(\phi - \theta\right)\right] \quad (17)$$

$$\eta_A = \frac{x_4}{2y} = 1 - \tan\theta \tan(\phi - \theta) \text{ for } \phi > \theta \quad (18)$$

$$\text{else } \eta_A = 1 \quad (19)$$

$$\eta_T = \frac{W}{W_0} = \varepsilon_A + 1 \quad (20)$$

$$\eta_O = \cos\gamma \quad (21)$$

where γ is the angle between the normal to the cell and the source:

$$\gamma = \cos^{-1}\left(\frac{a \cdot b}{|a||b|}\right) \quad (22)$$

where:

$$a = \begin{bmatrix} -\sin\phi\cos\alpha & \sin\phi\sin\alpha & \cos\phi \end{bmatrix} \quad (23)$$

and (for a panel with an E-W axis of rotation as was used in Phoenix, AZ during this study):

$$b = \begin{bmatrix} 0 & -\tan\theta & -1 \end{bmatrix} \quad (24)$$

Here, ϕ is the zenith angle, α is the azimuthal angle as measured clockwise from North, and θ the feature angle of the kirigami tracking structure.

Thus, for a kirigami tracking system with an E-W axis of rotation:

$$\eta_C = \eta_A \eta_T \eta_O = \left[1 - \tan\theta \tan(\phi - \theta)\right]_0^1 (\varepsilon_A + 1)\cos\gamma \quad (25)$$

For the case when the optical source is moving in the plane normal to the tracking axis (as per the figure above), cosine losses are solely dependent on θ and ϕ:

$$\eta_O = \cos(\phi - \theta) \quad (26)$$

$$\eta_C = \eta_A \eta_T \eta_O = \left[1 - \tan\theta \tan(\phi - \theta)\right](\varepsilon_A + 1)\cos(\phi - \theta) = (\varepsilon_A + 1)\sec\theta\cos\phi \quad (27)$$

where:

$$\theta = \cos^{-1}\left(\frac{1}{\varepsilon_A + 1}\right) \tag{4}$$

Upon simplifying, η_C may be written as:

$$\eta_C = \cos\phi\left(\varepsilon_A + 1\right)\left(\varepsilon_T + 1\right) \tag{28}$$
